# Small-Molecule Activators of Insulin-Degrading Enzyme Discovered through High-Throughput Compound Screening

**DOI:** 10.1371/journal.pone.0005274

**Published:** 2009-04-22

**Authors:** Christelle Cabrol, Malwina A. Huzarska, Christopher Dinolfo, Maria C. Rodriguez, Lael Reinstatler, Jake Ni, Li-An Yeh, Gregory D. Cuny, Ross L. Stein, Dennis J. Selkoe, Malcolm A. Leissring

**Affiliations:** 1 Department of Neuroscience, Mayo Clinic, Jacksonville, Florida, United States of America; 2 Department of Molecular Therapeutics, The Scripps Research Institute, Scripps Florida, Jupiter, Florida, United States of America; 3 Laboratory for Drug Discovery, Harvard NeuroDiscovery Center, Brigham & Women's Hospital and Harvard Medical School, Cambridge, Massachusetts, United States of America; 4 Center for Neurologic Diseases, Brigham and Women's Hospital and Harvard Medical School, Boston, Massachusetts, United States of America; Griffith University, Australia

## Abstract

**Background:**

Hypocatabolism of the amyloid β-protein (Aβ) by insulin-degrading enzyme (IDE) is implicated in the pathogenesis of Alzheimer disease (AD), making pharmacological activation of IDE an attractive therapeutic strategy. However, it has not been established whether the proteolytic activity of IDE can be enhanced by drug-like compounds.

**Methodology/Principal Findings:**

Based on the finding that ATP and other nucleotide polyphosphates modulate IDE activity at physiological concentrations, we conducted parallel high-throughput screening campaigns in the absence or presence of ATP and identified two compounds—designated Ia1 and Ia2—that significantly stimulate IDE proteolytic activity. Both compounds were found to interfere with the crosslinking of a photoaffinity ATP analogue to IDE, suggesting that they interact with a *bona fide* ATP-binding domain within IDE. Unexpectedly, we observed highly synergistic activation effects when the activity of Ia1 or Ia2 was tested in the presence of ATP, a finding that has implications for the mechanisms underlying ATP-mediated activation of IDE. Notably, Ia1 and Ia2 activated the degradation of Aβ by ∼700% and ∼400%, respectively, albeit only when Aβ was presented in a mixture also containing shorter substrates.

**Conclusions/Significance:**

This study describes the first examples of synthetic small-molecule activators of IDE, showing that pharmacological activation of this important protease with drug-like compounds is achievable. These novel activators help to establish the putative ATP-binding domain as a key modulator of IDE proteolytic activity and offer new insights into the modulatory action of ATP. Several larger lessons abstracted from this screen will help inform the design of future screening campaigns and facilitate the eventual development of IDE activators with therapeutic utility.

## Introduction

Alzheimer disease (AD) is a devastating and increasingly common neurodegenerative disorder characterized by abnormal accumulation of the amyloid β-protein (Aβ) in brain regions subserving memory and other cognitive functions [1]. Aβ is a complex mixture of peptides ranging in size from 37 to 43 amino acids that are cleaved from the amyloid precursor protein by the successive action of aspartyl proteases known as β- and γ-secretase [2]. A wealth of evidence from human molecular genetics, animal modeling studies and other fields supports the hypothesis that the proximal cause of AD is chronic elevations in cerebral Aβ, either all forms or specifically longer species, such as Aβ42, which have a greater propensity to self-assemble into neurotoxic oligomers and higher-order aggregates, and which predominate in the amyloid plaques that characterize the disease [3]–[6].

The latter findings have prompted extensive efforts to develop therapies aimed at achieving sustained reductions in cerebral Aβ, thereby reducing the formation of neurotoxic Aβ oligomers and plaques. Toward this common goal, several different strategies are being vigorously pursued, including the approaches of (i) lowering the production of total Aβ with secretase inhibitors, (ii) selectively lowering Aβ42 with γ-secretase modulators, (iii) antibody-based clearance of pre-existing Aβ deposits by means of active or passive immunization, and (iv) reducing the self-assembly of Aβ with anti-aggregation compounds [7]. An alternative approach that has received less attention is the strategy of increasing the proteolysis of Aβ after it is produced [8]. Accumulating evidence has shown that cerebral Aβ levels are potently regulated by the concerted action of several different proteases, including neprilysin and insulin-degrading enzyme (IDE) [8]. Overexpression of either of the latter proteases in AD mouse models has been shown to retard or even completely prevent amyloid plaque formation and downstream cytopathology [9], [10].

Notwithstanding the theoretical merits of this approach, the idea that Aβ degradation can be enhanced pharmacologically has generally been regarded as, at best, a challenging objective. Despite this widespread perception, this goal has in fact already been achieved in practice: Jacobsen and colleagues recently described the development of an inhibitor of plasminogen activator inhibitor-1, an endogenous inhibitor of the conversion of plasminogen to plasmin—a known Aβ-degrading protease—by tissue-type and urokinase-type plasminogen activators [11]. Notably, the inhibitor developed by this group was found to effectively lower brain Aβ levels and even to reverse cognitive defects in an AD mouse model [11].

In the present study, we sought to achieve this same goal, in this case by identifying compounds that activate or disinhibit IDE, an atypical zinc-metalloprotease that is strongly linked both functionally and genetically to the pathogenesis of AD [8], [12]–[14]. Among known Aβ-degrading proteases, several lines of evidence suggest that IDE is a particularly attractive target for pharmacological activation. First and foremost, a substantial body of genetic evidence implicates variations in and around the *Ide* gene with the incidence and onset of AD [15]–[18], thus strongly supporting a key role for this protease in disease-relevant functions. Second, several studies suggest that IDE—a secreted enzyme [19]—is the principal protease responsible for the degradation of Aβ in the extracellular space [20], [21]. Third, IDE possesses distinctive structural features that appear to render it uniquely amenable to pharmacological activation [22]. As recent crystal structures have revealed [23]–[25], IDE possesses an unusual tertiary structure, consisting of two bowl-shaped halves connected by a flexible linker. This configuration allows the enzyme to adopt an “open” conformation, that permits the entry of substrates and exit of products, and a “closed” conformation, wherein substrates are completely entrapped within an unusually large internal chamber [23]. Enzymologic and biophysical studies suggest that IDE normally exists in the closed, inactive conformation, held in place by a “latch” mechanism comprised of extensive hydrogen bonding between the N- and C-terminal halves [22], [23]. Mutations that disrupt this hydrogen bonding have been shown to activate the protease by as much as ∼40 fold, suggesting that the same effect might be achieved pharmacologically [22], [23]. Finally, multiple studies have suggested that IDE can be activated by any of several independent means, including substrate activation [26], [27] and cysteine-specific alkylation [28] through a variety of proposed mechanisms (see *Discussion*).

Of special relevance to the present application, IDE activity has also been shown to be modulated by ATP and other nucleotide polyphosphates in a manner that is both bidirectional and substrate-selective [24], [29], [30]. Camberos and colleagues initially reported that ATP inhibits IDE-mediated insulin degradation at physiological concentrations [30]. Remarkably, ATP and related compounds were later found to dramatically *activate* the hydrolysis of shorter substrates by as much as ∼70-fold [24], [29].

Prompted by the latter findings, here we conducted a high-throughput screening campaign optimized to detect compounds that interact with the putative ATP-binding domain within IDE. We discovered two compounds that activate IDE activity as much as ∼700%. Despite the lack of structural similarities with ATP, the discovered compounds induced similar enzymologic effects and competed with the crosslinking of an azido-ATP derivative to IDE, yet paradoxically were found to act synergistically with ATP in activating IDE proteolytic activity. We show further that the discovered compounds can activate the degradation of Aβ, albeit only when presented in a mixture also containing shorter substrates. Together, our study describes the discovery of the first drug-like activators of IDE, the characterization of which provides several new insights into the mechanistic basis for pharmacological activation of IDE.

## Results

**Figure 1:**
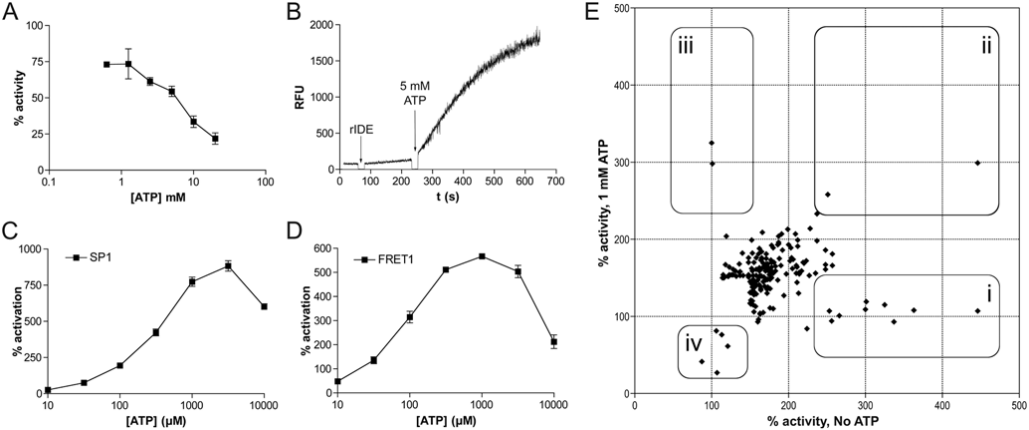
Assay development and rationale for parallel high-throughput IDE activator screening campaigns incorporating ATP. *A*, ATP inhibits IDE-mediated degradation of Aβ at physiological concentrations. *B*, Paradoxical ATP-mediated activation of the IDE-mediated hydrolysis of a fluorogenic peptide substrate. *C, D*, Dose-response of ATP on the hydrolysis of two structurally similar fluorogenic peptide substrates, SP1 (*C*) and FRET1 (*D*). Note the different degrees of maximal activation obtained for SP1 *vs*. FRET1. See Fig. S1 for a comparison of the structures of SP1 and FRET1. *E*, A subset of primary screening data showing percent activity in the presence *vs*. the absence of ATP (1 mM). *Rounded rectangles* show different categories of hits that can theoretically be identified using this approach, including (*i*) compounds that mimic ATP, (*ii*) compounds that activate IDE independently of ATP, (*iii*) compounds that act synergistically with ATP, and (*iv*) compounds that disrupt the activation effect induced by ATP. Data are mean±SEM for 4 to 5 independent experiments each comprising 3 to 4 replicates and normalized to control wells containing no ATP. For panels *A*, *C* and *D*, all data for concentrations of ATP above 10 µM are significantly different from the no-ATP controls (*P*<0.05).

Toward the goal of discovering pharmacological activators of IDE, we planned a compound screening campaign using the fluorogenic peptide substrate, SP1 (Fig. S1A), as a read-out for IDE activity. The goal of identifying activators of a protease is greatly facilitated if its activity is normally regulated by endogenous inhibitors, for this makes it possible to identify compounds that disrupt the inhibitor-protease interaction, thereby disinhibiting—*i.e.*, activating—the protease. Based on a report showing that insulin degradation by IDE was inhibited by ATP [30], we therefore planned to conduct parallel screening campaigns incorporating this putative endogenous inhibitor. During the assay development phase, we confirmed that ATP inhibited the degradation of Aβ by IDE at physiological concentrations (Fig. 1A). Unexpectedly, ATP was found to potently activate the hydrolysis of SP1 (Fig. 1B,C; [31]) and also a structurally similar fluorogenic peptide substrate, FRET1 (Fig. 1D; Fig. S1B; [28]), a result that was confirmed by subsequent reports [24], [29]. Based on these results, we conducted parallel compound screening campaigns in the presence or absence of a near-maximal activating concentration of ATP (1 mM; see *Materials and Methods*). By comparing the effects of compounds under these contrasting conditions (Fig. 1E), several categories of compounds could in principle be identified, including compounds that mimic ATP (Fig. 1E,i), compounds that activate IDE independently of ATP (Fig. 1E,ii), compounds that act synergistically with ATP (Fig. 1E,iii), and compounds that disrupt the activation effect induced by ATP (Fig. 1E,iv).

The primary screening campaign was conducted robotically in 384-well format using a library of ∼32,000 compounds comprised of 704 FDA-approved compounds, 352 natural products, a tetrapeptide library consisting of all possible permutations of 8 amino acids (Glu, His, Lys, Pro, Gln, Val, Trp, Tyr), and an additional 27,300 small-molecule pharmacophores conforming to Lipinski's rules [32]. Compounds showing activation >150% in the absence or presence of ATP were cherry-picked, spotted on 384-well plates and rescreened robotically. For compounds showing reproducible activation, dose-response curves were subsequently determined manually using both SP1 and FRET1, and for this phase, the fluorescence read-out was monitored continuously throughout the reaction period to identify compounds exhibiting fluorescence artifacts.

**Figure 2:**
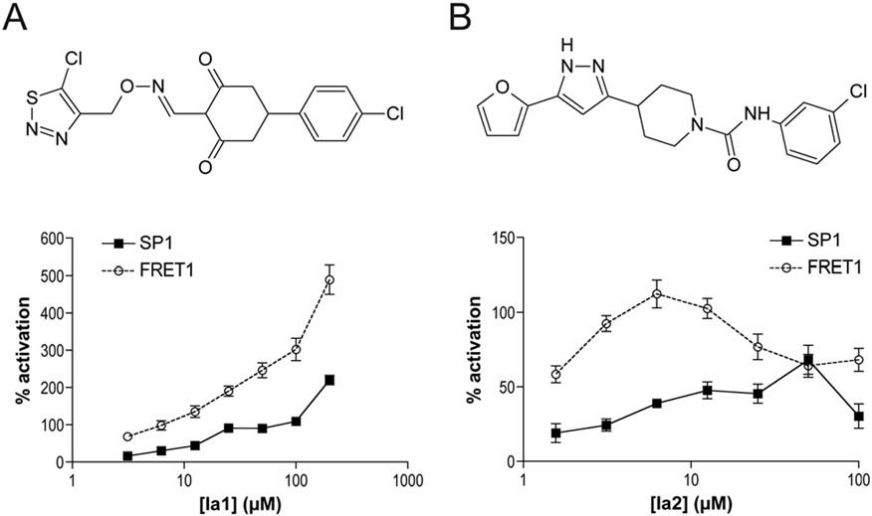
Small-molecule activators of IDE discovered through compound screening. *A*,*B*, Structures of Ia1 (*A*) and Ia2 (*B*) and dose-dependent effects on the IDE-mediated hydrolysis of SP1 and FRET1. Data are mean±SEM for 4 to 5 independent experiments normalized to control wells containing no ATP. Data for all doses showing ≥30% activation are statistically different from DMSO controls (*P*<0.05).

Two compounds that consistently activated IDE—dubbed Ia1 and Ia2 (IDE activators 1 and 2; LDN-1487 and LDN-1844, respectively)—emerged from the screening campaign (Fig. 2A,B). Ia1 activated the hydrolysis of SP1 and FRET1 by up to ∼200% and ∼500%, respectively, in a dose-dependent manner, with maximal activation occurring at 200 µM (Fig. 2A). Ia2 also activated both substrates, albeit to a lesser extent: maximal activation by Ia2 was determined to be ∼110% and ∼60% at concentrations of 50 µM and 6.25 µM, for SP1 and FRET1, respectively.

A small number of compounds structurally related to Ia2 were either present in the compound library or available commercially, allowing the structure-activity relationship to be investigated. These compounds all contained 4-[5-furan-2-yl)-1*H-*pyrazol-3-yl]piperidine, with different substitutions at the piperidine nitrogen functionality (Fig. 2A,B). When tested at 10 µM, the percent activation produced by different substitutions followed the series: -H<-CH_2_NC≈(4-methylphenyl)sulfonyl≤-CONH-3-chlorophenyl, with maximal activation produced by Ia2 itself (Fig. S2).

**Figure 3:**
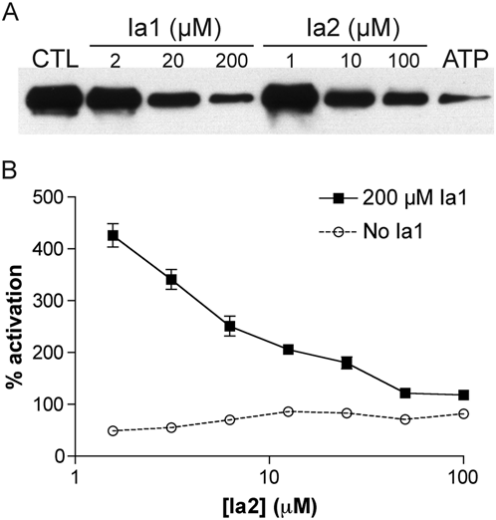
Ia1, Ia2 and ATP interact with a common domain within IDE. *A*, Ia1 and Ia2 disrupt the crosslinking of recombinant IDE with a photoaffinity ATP analogue. DMSO concentrations were maintained constant in all conditions; *CTL* = DMSO only; *ATP* = 10 mM ATP. *B*, Activation of FRET1 hydrolysis by Ia1 can be competed away with increasing doses of Ia2. Data are mean±SEM for 5 independent experiments normalized to control wells containing DMSO only. All data are statistically different from controls (*P*<0.05).

Although Ia1 and Ia2 and its variants show no obvious structural (Fig. S3) or chemical (Table S1) relationship to ATP or other nucleotide polyphosphates, their similar size and common ability to activate IDE suggested that they may exert their effects by interacting with a common domain within IDE. To investigate this hypothesis, we tested the ability of Ia1 and Ia2 to interfere with the binding of ATP, as determined from the labeling of recombinant IDE with a biotinylated photoaffinity ATP analogue, 8-azidoadenosine 5′-triphosphate 2′,3′-biotin-LC-hydrazone (azido-ATP-biotin). Under conditions that produced substantial photocrosslinking of IDE, Ia1 and Ia2 both interfered with azido-ATP-biotin labeling in a dose-dependent manner, an effect that was also produced by excess unlabeled ATP, as expected (Fig. 3A). To investigate the interaction of Ia1 and Ia2 with one another, we tested and confirmed the ability of Ia2 to interfere with the more pronounced activation effect produced by Ia1 (Fig. 3B). Together, these results support the idea that Ia1 and Ia2 both exert their effects through interaction with a common domain within IDE that also binds to ATP.

**Figure 4:**
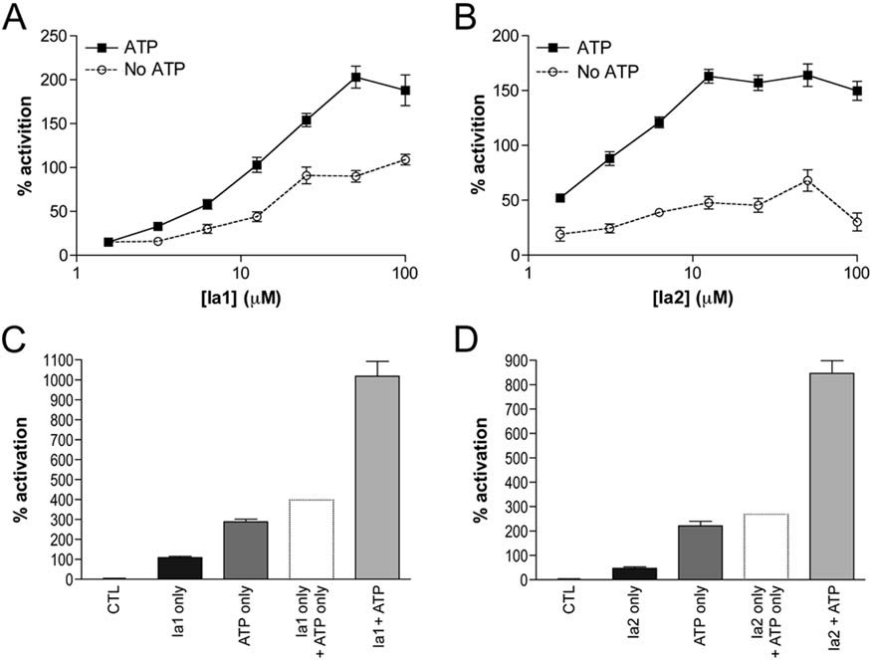
Synergistic effects of ATP and Ia1 or Ia2. *A*,*B*, Activating effects of Ia1 (*A*) and Ia2 (*B*) on IDE-mediated hydrolysis of SP1 when tested in the absence or presence of ATP (0.1 mM). Data have been normalized to controls containing DMSO only or DMSO plus ATP, respectively. *C*,*D*, Pronounced synergy between ATP and Ia1 (50 µM; *C*) and Ia2 (10 µM; *D*). In this case, data are presented as raw activation values, all normalized to DMSO-only controls lacking ATP. *Dashed columns* show the predicted magnitude of activation if the effects of ATP and Ia1 or Ia2 were simply additive. Data are mean±SEM for 4 independent experiments. For panels *A* and *B*, data showing ≥30% activation are statistically different from corresponding controls (*P*<0.05). For panels *C* and *D*, data are statistically different for all comparisons (*P*<0.01).

To further explore the functional interrelationship among ATP and the identified IDE-activating compounds, we determined the degree of activation produced by different doses of Ia1 and Ia2 in the absence or presence of a partially activating concentration of ATP (0.1 mM). Based on the preceding analysis we expected that the identified compounds would, depending on their relative binding affinities and activating potential, either block the activating effect of ATP or have little or no additional activating effect. Instead, when SP1 was used as a substrate, a pronounced synergistic activation effect was observed between ATP and both Ia1 and Ia2 (Fig. 4), increasing IDE activity to a degree not achieved with ATP alone (*e.g.*, to ∼1000% for Ia1 and ATP; *c.f.* Fig. 1C). A similar interaction between ATP and the activating compounds was observed for FRET1 (not shown).

**Figure 5:**
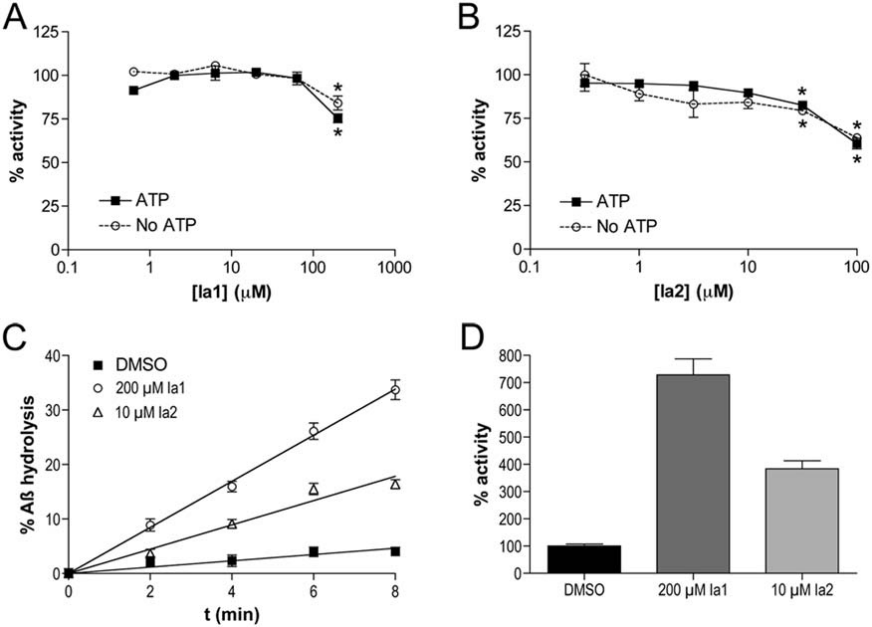
Effects of Ia1 and Ia2 on Aβ degradation. *A*,*B*, Ia1 (*A*) and Ia2 (*B*) fail to activate the degradation of Aβ, in the presence or the absence of ATP, when Aβ is the sole substrate; Instead, partial inhibition is seen at higher concentrations. Data are mean±SEM for 4 independent experiment; *P<0.05. *C*,*D*, Ia1 and Ia2 strongly activate Aβ degradation when presented together with a short substrate (FRET1; 10 µM). *C*, Representative time-course of Aβ activation by Ia1 and Ia2. *D*, Results of 4 independent experiments showing results normalized to DMSO-only controls. Data are mean±SEM and are statistically different for all comparisons (*P*<0.01).

We then explored the effects of Ia1 and Ia2 on the degradation of Aβ by IDE. To the extent that the IDE-activating compounds mimic the actions of ATP, they were predicted to exert no effect or even to inhibit IDE-mediated Aβ degradation. Alternatively, however, they might prove capable of relieving the inhibitory effect of ATP on Aβ degradation (see Fig. 1A), thereby activating the protease. To explore these possibilities, we tested the effects of Ia1 and Ia2 on IDE-mediated Aβ degradation in the absence or presence of a partially inhibitory concentration of ATP (5 mM). With the exception of very high doses of Ia1 or Ia2, which elicited partial inhibition, we observed no significant effect of Ia1 and Ia2 on Aβ degradation in the presence or absence of ATP (Fig. 5A,B).

Although Ia1 or Ia2 failed to activate the degradation of Aβ by itself, we reasoned that activation may yet occur when Aβ is present in a more complex mixture also containing shorter substrates whose hydrolysis can be affected by the activating compounds. To test this idea, we monitored the degradation of Aβ mixed together with FRET1. Under these conditions, Ia1 and Ia2 were both found to produce significant activation of IDE-mediated Aβ degradation (Fig. 5C,D). Similar effects were observed in the presence of SP1 (not shown). It is notable that the degree of activation of Aβ degradation induced by Ia1 and Ia2 under these conditions exceeded that achieved with FRET1 or SP1 alone (*c.f.* Figs. 2 and 5C,D).

## Discussion

In the present study we conducted a high-throughput compound screening campaign optimized to discover compounds that interact with a putative ATP-binding domain within IDE. We succeeded in identifying two compounds, Ia1 and Ia2, that significantly stimulate IDE proteolytic activity, which were characterized extensively *in vitro*. To our knowledge, Ia1 and Ia2 are the first drug-like compounds (*i.e.*, conforming to Lipinski's rules [32]) shown to activate this important protease.

Despite the lack of structural or chemical similarities to ATP or other nucleotide polyphosphates, Ia1 and Ia2 both exhibited largely similar enzymologic properties as the latter molecules. Like nucleotide polyphosphates, Ia1 and Ia2 both stimulated the proteolysis of short peptide substrates, but failed to affect full-length Aβ when presented as the sole substrate. Significantly, both of the IDE-activating compounds we identified were capable of blocking the crosslinking of IDE with a photoaffinity ATP analogue, and the activating effect of one compound, Ia1, could be blocked by the more weakly activating compound, Ia2. Taken together, these results suggest that ATP, Ia1 and Ia2 all interact with a common domain within the IDE molecule.

The latter conclusion, however, is seemingly contradicted by the fact that Ia1 and Ia2 were each found to act *synergistically* with ATP, notably activating IDE to a degree not achieved with ATP, Ia1 or Ia2 alone. One possibility is that Ia1 and Ia2 bind to a separate, perhaps partially overlapping site, which in turn affects ATP binding. Under this scenario, it is possible that the activating compounds may only subtly affect the positioning of ATP, rather than its binding *per se*, which could in turn influence the ability of azido-ATP-biotin to crosslink to IDE.

Another important observation that may also help to explain this apparent discrepancy is the finding that IDE can be activated not only by nucleotide triphosphates, but also by inorganic triphosphate (PPPi) alone [24], [29]. Despite similar activation effects, dissociation has been observed between the effects of ATP and PPPi in two important respects. First, ATP, but not PPPi, was found to slow the electrophoretic mobility of IDE under native conditions [24]. Second, ATP, but not PPPi, was found to induce significant changes in the secondary structure of IDE, as determined from circular dichroism experiments [24]. The latter results indicate that PPPi, possibly by virtue of its highly polar nature, can induce effects that are independent of the ability of the full-length ATP molecule to bind to IDE. Thus, it seems plausible that the synergistic effects observed between ATP and Ia1 or Ia2 could be attributable to an interaction between their binding to a *bona fide* nucleotide polyphosphate-binding domain within IDE combined with non-specific effects induced by the highly charged triphosphate moiety within ATP.

The preceding conclusion is further supported by the fact that multiple, distinct mechanisms have been proposed to underlie activation of ATP by IDE. One proposed mechanism points to effects on IDE's quaternary structure. IDE normally exists as a homodimer, but can also assemble into higher n-oligomers, the formation of which inhibits IDE activity [23], [29]. Accordingly, gel filtration experiments have demonstrated that ATP suppresses higher n-oligomer formation [29]. A second mechanism suggests that ATP may act by influencing the transition between the “open” configuration of IDE and its “closed”, inactive conformation. This model is supported by the observation that ATP can induce increases in the hydrodynamic radius of IDE as detected by dynamic light scattering [24]. The observed synergy between ATP and Ia1 or Ia2 might therefore be explained if either or both of the latter mechanisms are induced by the triphosphate moiety within ATP, but not by Ia1 or Ia2 alone.

If the aforementioned mechanisms apply only to effects induced by the triphosphate moiety of ATP, what mechanism(s) underlies the effects of Ia1, Ia2, and, by extension, nucleotide polyphosphates, when bound to the domain(s) with which they interact? Though purely speculative, an attractive possibility is that these molecules all bind to one or more sites located within the internal chamber of IDE. Such binding would be predicted to decrease the volume accessible to substrate, thereby reducing the degrees of freedom available for its positioning and so increasing the probability that it will adopt a proteolysis-competent conformation (see [25]). This model might also help to explain why ATP (and Ia1 and Ia2 at high concentrations) inhibits the hydrolysis of larger substrates such as insulin or Aβ: by virtue of their larger size, these substrates might be incapable of occupying the internal chamber together with ATP or the activating compounds we identified.

In terms of potential binding domains within IDE, it is notable that an exosite has been identified within IDE's internal chamber [23]–[25]. Crystal structures of IDE show that this domain is located at the opposite side of the internal chamber as the active site, is involved in the binding of larger substrates, and shows a propensity to bind smaller peptides, as well [23]–[25]. While it remains plausible that Ia1, Ia2 and ATP exert their action through binding to this domain, we note that the screening campaign included 4096 tetrapeptides, none of which exhibited activation effects comparable to Ia1 or Ia2, either in the presence or absence of ATP.

An important and unanticipated finding of the present study is the observation Ia1 and Ia2 significantly activated the degradation of Aβ when presented in a mixture together with a shorter substrate, though not when Aβ was presented alone. Significantly, the degree of activation of Aβ degradation under the former conditions was larger than observed for the shorter substrate by itself. Mechanistically, we speculate that this phenomenon may be related to positive intersubunit cooperativity conferred by the homodimeric configuration of IDE. Under this scenario, the interaction of one subunit with substrate may render the other subunit more capable of binding substrate (or releasing products) through allosteric effects. Such an effect might be more pronounced for combinations of different substrates than for a single substrate presented alone. At a more practical level, this result contains an important lesson for future compound screening campaigns: whereas it is commonly considered desirable to utilize the most reductionistic arrangement possible, our results suggest that screening with a single substrate may not be ideal in the case of IDE. Instead, it may be preferable to screen under conditions that contain a complex mixture of substrates, ideally mimicking as closely as possible the substrate milieu in which therapeutic compounds are eventually intended to act. Indeed, given IDE's unusual structure, mode of substrate processing and sensitivity to endogenous cofactors [23], it may ultimately be possible to identify compounds that operate in one physiological context but not others (*e.g.*, intracellular vs. extracellular).

By way of conclusion, we point out that ATP is just one of several candidate endogenous inhibitors of IDE. Other attractive candidates include ubiquitin [33] and long-chain fatty acids [34]. The general strategy we employed—namely, conducting parallel screens in the presence or absence of the putative endogenous inhibitor—appears to be ideal for reliably identifying compounds that displace endogenous inhibitors and for rapidly distinguishing them from compounds acting by alternative mechanisms.

## Materials and Methods

### Chemicals

The compound library used for screening was comprised of commercially available compounds purchased from Peakdale (High Peak, UK), Maybridge Plc. (Cornwall, UK), Cerep (Paris, France), Bionet Research Ltd. (Cornwall, UK), Prestwick (Ilkirch, France), Specs and Biospecs (CP Rijswijk, the Netherlands), ENAMINE (Kiev, Ukraine), and ChemDiv (San Diego, CA). Ia1 and Ia2 were purchased from Bionet. SP1 was synthesized by SynPep Corp. (Dublin, CA). FRET1 was synthesized by Margaret Condron and David Teplow as described [28]. Azido-ATP-biotin was purchased from ALT Bioscience (Lexington, KY). All other chemicals and reagents were purchased from Sigma-Aldrich Co. (St. Louis, MO).

### High-throughput compound screening

Compounds were spotted robotically onto black 384-well plates (0.4 µL/well, 50 µM final concentration) and stored at −80°C. On the day of screening, plates were thawed, then reagents dissolved in reaction buffer (50 mM Tris HCl, pH 7.4 supplemented with 0.1% BSA) were added robotically using a BioMek FX Laboratory Automation Workstation (Beckman-Coulter) under either of two conditions. For experiments carried out in the absence of ATP, recombinant IDE (30 µL/well, 6 nM final concentration) was added robotically, agitated on an orbital shaker for 1 min to facilitate mixing, then allowed to equilibrate with compounds for 1 h at room temperature. The reaction was then initiated by addition of SP1 (6 µM final concentration; 10 µL/well), agitated for 1 min, then terminated 4 h later by addition of stop buffer (0.1 M NaOAc, pH 4.0; 10 µL/well). For experiments carried out in the presence of ATP, the recombinant IDE solution was supplemented with ATP (1 mM final concentration) and the reaction time was adjusted to 1.5 h. Controls added to each assay plate included wells lacking IDE (0% activity), recombinant IDE supplemented with DMSO (0.4 µL/well), recombinant IDE supplemented with the competitive inhibitor, insulin (10 µM final concentration), and excess IDE (20 nM final concentration; 100% activity. Activity was assessed by quantification of fluorescence intensity (λ_ex_ 330 nm, λ_em_ 450 nm) using an Analyst HT multilabel plate reader (Molecular Devices) equipped with integrated stackers. To minimize the influence of the intrinsic fluorescence of compounds, fluorescence was quantified immediately after termination of the reaction and subtracted from the fluorescence measurement determined after addition of substrate. Raw fluorescent data were converted to % activity by normalization to intra-plate controls. For replication experiments, compounds were cherry-picked from thawed DMSO stocks, spotted manually onto assay plates and processed as above.

### Dose-response determinations

For compounds showing reproducible activation, DMSO stocks were cherry-picked, diluted in DMSO, spotted manually onto assay plates, then assayed as above, albeit using manual solution transfers. For these experiments, the reaction was monitored continuously by determining fluorescence intensity (λ_ex_ 330 nm, λ_em_ 420 nm) at 2-min intervals using a Gemini XL multilabel plate reader (Molecular Devices).

### Enzymologic studies

Compounds selected for further *in vitro* characterization were purchased from commercial vendors as lyophilized stocks, resuspended in DMSO, and stored in aliquots at −80°C. Assays incorporating fluorogenic substrates SP1 and FRET1 (5 µM) were conducted essentially as above using a SpectraMax M5 multilabel plate reader (Molecular Devices). Degradation of Aβ (50 nM) was quantified as described [35].

### Photoaffinity labeling

Photocrosslinking with azido-ATP-biotin was conducted according to manufacturer's recommendations (ALT Bioscience). Briefly, the ATP analogue dissolved in MeOH was added to 0.2 mL tubes (5 nmol/tube), and MeOH was removed by lyophilization. The ATP analogue was dissolved in a solution containing 20 nM recombinant IDE (10 mM HEPES pH 7.4), then reactions were exposed to UV radiation (254 nm, 5 mW/cm^2^) for 2 min using a hand-held lamp and terminated by addition of dithiothreitol (to 10 mM final concentration). The terminated reactions were processed by conventional SDS-PAGE under denaturing and reducing conditions and transferred to nitrocellulose membranes as described [9]. Membranes were blocked in 5% non-fat milk in Tris-buffered saline supplemented with 0.2% Tween-20 (TBS-T), incubated for 1 h with anti-biotin monoclonal antibody, BN-34 (1∶1000; Sigma), washed in TBS-T, incubated with a peroxidase-conjugated anti-mouse IgG secondary antibody (1∶5000, Vector Labs), washed, then detected by enhanced chemoluminescence.

## Supporting Information

Figure S1 Chemical structures of SP1 and FRET1.
(0.07 MB TIF)

Figure S2 Structure-activity relationships for variants of Ia2. A, Domain within Ia2 that is common to all variants. B, Activity (left) of Ia2 variants containing different substitutions (right).
(0.09 MB TIF)

Figure S3 Chemical structures of Ia1 (A), Ia2 (B) and ATP (C).
(0.09 MB TIF)

Table S1 Comparison of selected chemical properties of Ia1, Ia2 and ATP.
(0.03 MB DOC)
