# Supplementary figures and images for: Small-Molecule Activators of Insulin-Degrading Enzyme Discovered through High-Throughput Compound Screening
Source: PLoS One. 2009 Apr 22;4(4):e5274. doi: 10.1371/journal.pone.0005274 (PMC2668070; doi:10.1371/journal.pone.0005274)

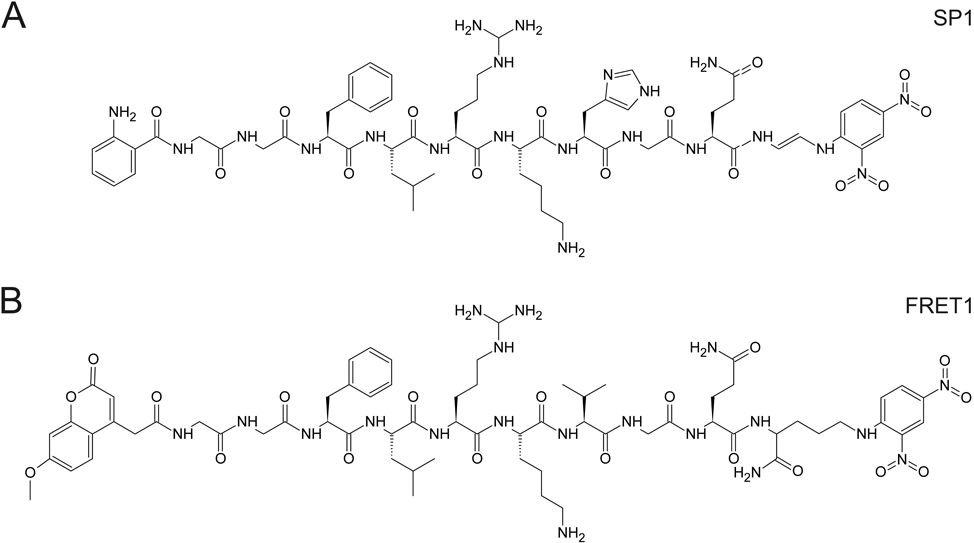

Supplement: Figure S1 — Chemical structures of SP1 and FRET1. (0.07 MB TIF) [file pone.0005274.s001.tif]

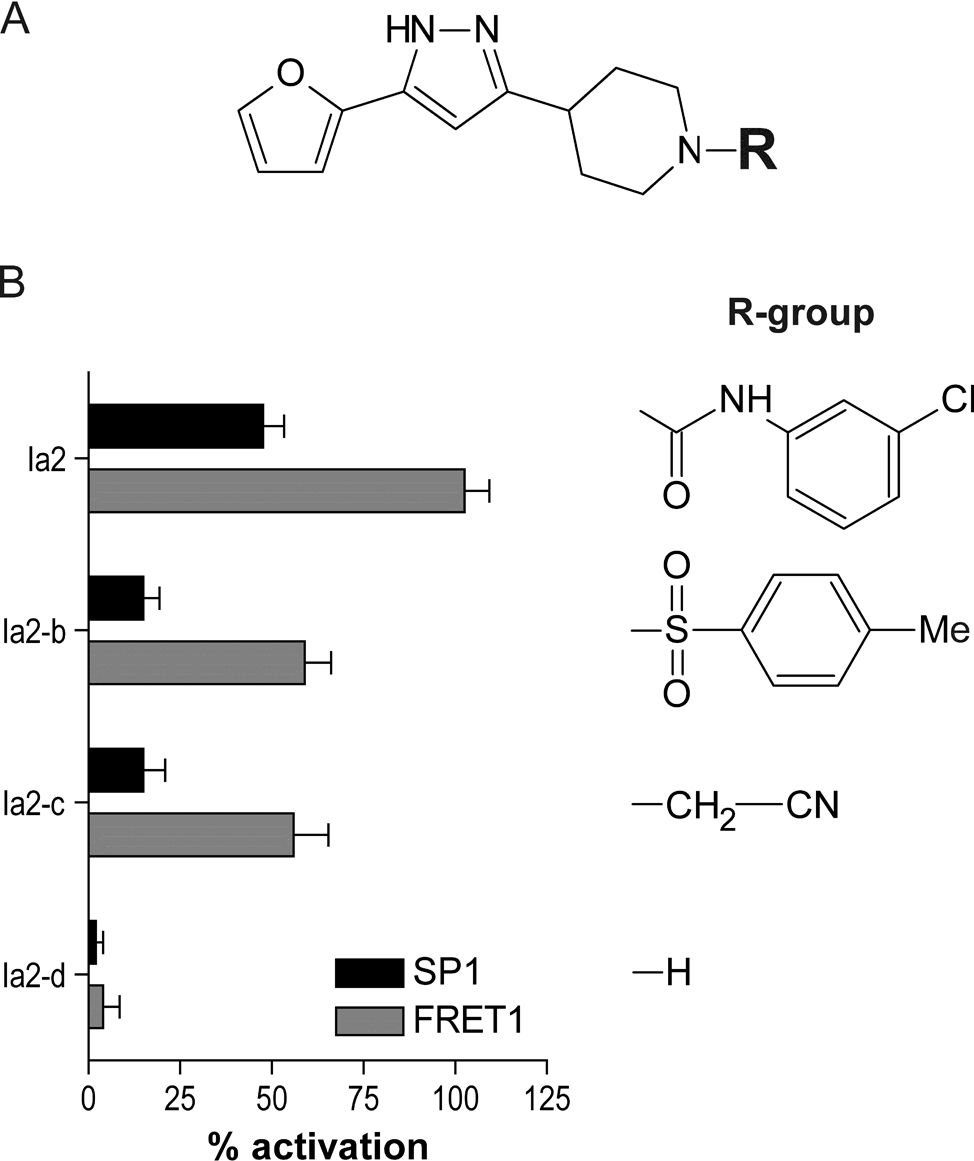

Supplement: Figure S2 — Structure-activity relationships for variants of Ia2. A, Domain within Ia2 that is common to all variants. B, Activity (left) of Ia2 variants containing different substitutions (right). (0.09 MB TIF) [file pone.0005274.s002.tif]

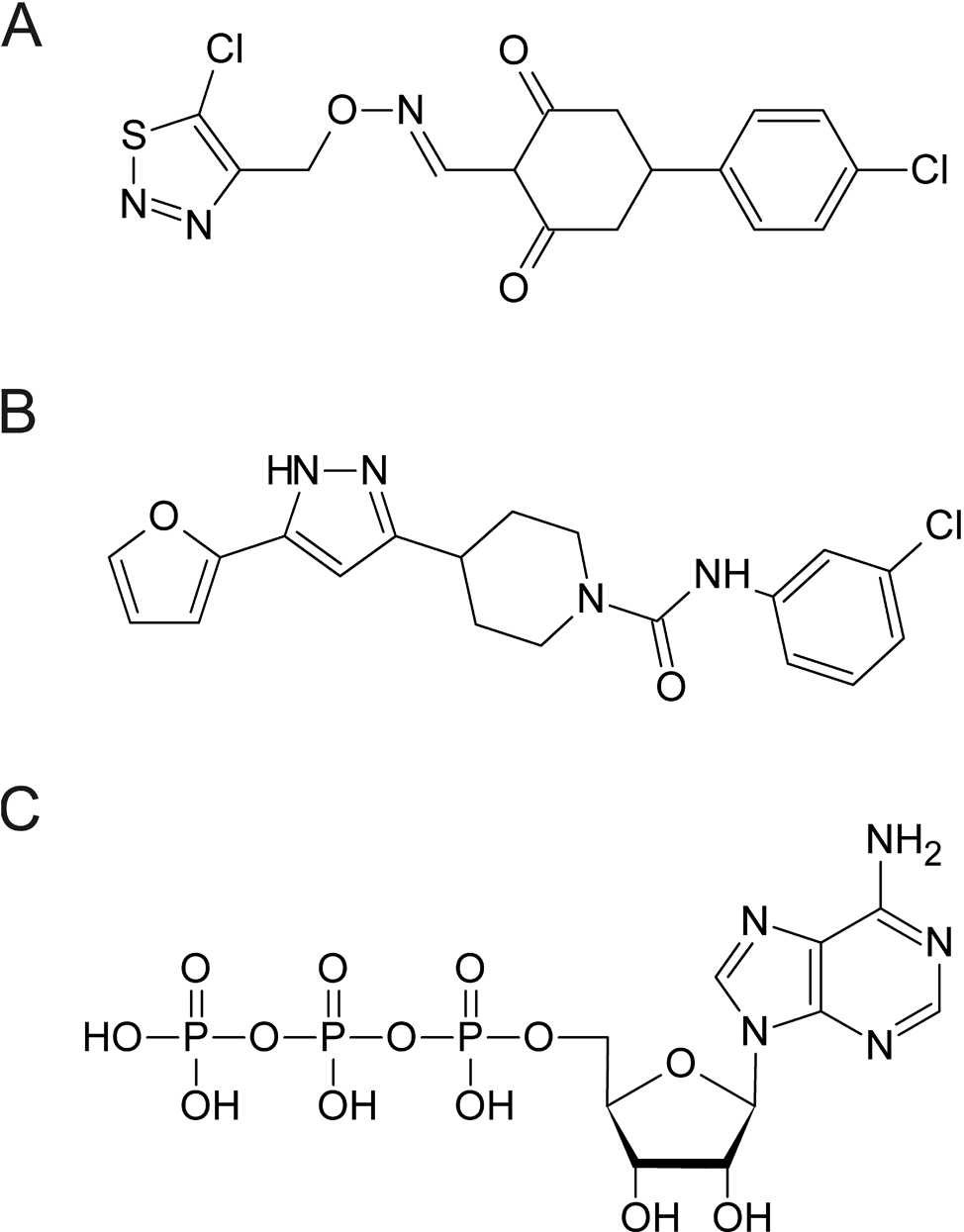

Supplement: Figure S3 — Chemical structures of Ia1 (A), Ia2 (B) and ATP (C). (0.09 MB TIF) [file pone.0005274.s003.tif]
