# Supplementary material for: Small-Molecule Activators of Insulin-Degrading Enzyme Discovered through High-Throughput Compound Screening
Source: PLoS One. 2009 Apr 22;4(4):e5274. doi: 10.1371/journal.pone.0005274 (PMC2668070; doi:10.1371/journal.pone.0005274)
Supplement: Table S1 — Comparison of selected chemical properties of Ia1, Ia2 and ATP. (0.03 MB DOC) [file pone.0005274.s004.doc]

**Table S1.** Comparison of selected chemical properties of Ia1, Ia2 and ATP.

| Compound: | Ia1 | Ia2 | ATP |
| --- | --- | --- | --- |
| PubChem ID | 1488670 | 5934897 | 5957 |
| Molecular weight (g/mol) | 370.83 | 398.26 | 507.18 |
| Empirical Formula | C_16_H_13_C_l2_N_3_O_3_S | C_19_H_19_ClN_4_O_2_ | C_10_H_16_N_5_O_13_P_3_ |
| XlogP | 3.8 | 3.3 | -5.5 |
| H-bond donors | 0 | 2 | 7 |
| H-bond acceptors | 6 | 4 | 18 |
